# Lateralized Contributions of Medial Prefrontal Cortex Network to Episodic Memory Deficits in Subjects With Amnestic Mild Cognitive Impairment

**DOI:** 10.3389/fnagi.2021.756241

**Published:** 2021-11-17

**Authors:** Qing Ye, Haifeng Chen, Renyuan Liu, Ruomeng Qin, Caimei Luo, Mengchun Li, Yun Xu, Hui Zhao, Feng Bai

**Affiliations:** ^1^Department of Neurology, Nanjing Drum Tower Hospital, Nanjing University Medical School, Nanjing, China; ^2^The State Key Laboratory of Pharmaceutical Biotechnology, Institute of Brain Science, Nanjing University, Nanjing, China; ^3^Jiangsu Province Stroke Center for Diagnosis and Therapy, Nanjing, China; ^4^Nanjing Neuropsychiatry Clinic Medical Center, Nanjing, China; ^5^Department of Radiology, Nanjing Drum Tower Hospital, Nanjing University Medical School, Nanjing, China

**Keywords:** episodic memory, executive function, mild cognitive impairment, functional magnetic resonance imaging, hippocampal network, medial prefrontal cortex network

## Abstract

Both episodic memory and executive function are impaired in amnestic mild cognitive impairment (aMCI) subjects, but it is unclear if these impairments are independent or interactive. The present study aimed to explore the relationship between episodic memory deficits and executive function deficits, and the underlying functional mechanisms in aMCI subjects. Thirty-one aMCI subjects and 27 healthy subjects underwent neuropsychological tests and multimodal magnetic resonance imaging (MRI) scans. Hippocampal networks and medial prefrontal cortex (MPFC) networks were identified based on resting-sate functional MRI (fMRI) data. AMCI subjects displayed lower episodic memory scores and executive function scores than control subjects, and the episodic memory scores were positively correlated with the executive function scores in aMCI subjects. Brain network analyses showed an interaction between the hippocampal networks and the MPFC networks, and the interaction was significantly associated with the episodic memory scores and the executive function scores. Notably, aMCI subjects displayed higher functional connectivity (FC) of the right hippocampal network with the right prefrontal cortex than did control subjects, but this difference disappeared when controlling for the MPFC networks. Furthermore, the effects of the MPFC networks on the hippocampal networks were significantly associated with the episodic memory scores in aMCI subjects. The present findings suggested that the episodic memory deficits in aMCI subjects could be partially underpinned by the modulation of the MPFC networks on the hippocampal networks.

## Introduction

Alzheimer’s disease (AD) is usually characterized by episodic memory deficits and impairment in other cognitive domains, including executive function, language, and visuospatial function. Patients with amnestic mild cognitive impairment (aMCI), a transitional stage between normal aging and AD, have a high risk of developing AD. These patients develop AD at a rate of 10–15% per year (Petersen and Negash, 2008). Episodic memory and executive function are also impaired in aMCI patients (Kirova et al., 2015).

Executive function is broadly defined as control processes responsible for planning, assembling, coordinating, sequencing, and monitoring other cognitive operations. Many studies showed that executive function was consistently linked to episodic memory performance both in childhood and old age (Davidson and Glisky, 2002; Park et al., 2002; Dias et al., 2018). The executive decline hypothesis suggests that the effect of aging on memory may be due to a subclinical and selective decline in the specific executive functions listed above (Salthouse et al., 2003). In functional anatomy, the hippocampus plays a key role in episodic memory processing, and the medial prefrontal cortex (MPFC) serves as a central component of systems involved in executive processing (Leh et al., 2010; Brown, 2011). Both the hippocampus and the prefrontal lobe are vulnerable to damages induced by aging or aging-related disease (Moscovitch and Winocur, 1995; West, 1996; Wu et al., 2008). Initial pathological changes in AD are found in the medial temporal lobe, especially the hippocampus. Other regions, including the MPFC, are affected as the disease progresses (Nordahl et al., 2005). Therefore, it is not surprising that aMCI patients typically have prominent episodic memory deficits. On the other hand, executive decline has also been observed in aMCI patients (Yoon et al., 2013), and the impairment of executive function is associated with a high rate of progression from mild cognitive impairment to dementia (Aretouli et al., 2013). It is largely unclear if episodic memory loss and executive decline are independent or interactive in aMCI patients.

A specific cognitive function is controlled by a distributed neural network rather than a specific brain region. The hippocampus interacts extensively with a number of specific distributed cortical and subcortical structures in frontal lobe, parietal lobe and basal ganglia to support episodic memory (Moscovitch et al., 2016). The MPFC is a vital component for episodic memory and is also responsible for executive function. The posterior cortical regions and subcortical structures interact with the prefrontal cortex to mediate successful executive processing (Funahashi and Andreau, 2013). There appears to be overlapping brain regions that, at least partially, support both executive function and episodic memory processing. Interestingly, effects of white matter hyperintensities and hippocampal volume loss on episodic memory in aMCI patients and demented patients can be mediated by executive functions, as evidenced by structural magnetic resonance imaging (MRI) data (Parks et al., 2011). Executive function also mediates age-related changes in episodic memory in elderly population (Lee et al., 2012).

Recently, advances in the development and application of functional MRI (fMRI) allow us to investigate the topological patterns of human brain networks *in vivo*. Interactions between medial temporal lobe and prefrontal cortex contributing to normal memory function have been shown by neuroimaging studies (Simons and Spiers, 2003; Dickerson et al., 2007). Activities in medial temporal lobe and prefrontal cortex during encoding predict the memory performance in cognitively normal adults (Dickerson et al., 2007). Previous fMRI studies on aMCI subjects have shown an altered medial temporal lobe in hippocampal networks (Risacher et al., 2013; Liu et al., 2016; Ye et al., 2017) and altered prefrontal cortex in executive control networks (Liu et al., 2012). Lateralized activation in the hippocampus and the altered functional connectivity (FC) of the hippocampal network with the right prefrontal cortex have been shown to regulate memory function (Anderson et al., 2004; Ye et al., 2017). However, less study has directly examined possible interactions between these functional networks in aMCI subjects. In this study, we utilized neuropsychological testing and the fMRI technology to investigate the cognitive function and the network organization of the MPFC networks and the hippocampal networks in aMCI subjects and healthy subjects. We tested the hypothesis that episodic memory deficits in aMCI may be partially due to impaired executive function. We further tested whether this hypothesized effect could be explained with lateralized communication between the MPFC networks and the hippocampal networks.

## Materials and Methods

### Subjects

Fifty-eight subjects (all right-handed), including 31 aMCI subjects and 27 healthy controls were recruited through community health screening and newspaper advertisements. The present study was carried out in accordance with the latest version of the Declaration of Helsinki, and approved by the Drum Tower Hospital Research Ethics Committee. Written informed consent was provided from each subject.

### Neuropsychological Examination

Global cognitive function was measured using a Mini Mental State Examination (MMSE) and a Clinical Dementia Rating (CDR). All subjects underwent a neuropsychological battery test including modified auditory-verbal learning test (AVLT) for Chinese, visual reproduction-delayed recall (VR-DR), Trail Making Tests (TMT)-A and -B, Stroop Color and Word Test-A, -B, and -C (Stroop-A, -B and -C), Clock Drawing Test (CDT), and Boston Naming Test (BNT). The modified AVLT included three auditory verbal learning test-immediate recalls (AVLT-IR), two auditory-verbal learning test-delayed recalls (AVLT-DR) and one delayed recognition (Guo et al., 2009). The AVLT-IRs are associated with encoding, while the last AVLT-DR is associated with consolidation and retrieval (Delis et al., 1991; Lueke and Lueke, 2019). Two aMCI subjects failed to perform some of tests due to subjective unwillingness or vision deterioration. The mental statuses were assessed with the Structured Clinical Interview for Diagnostic and Statistical Manual of Mental Disorders, Fourth Edition (DSM-IV) Axis I Disorders, the Hamilton Anxiety Scale (HAMA), and the Hamilton Depression Scale (HAMD).

### Inclusion and Exclusion Criteria

The diagnosis of aMCI was made following the recommendations of Petersen et al. (1999) and Winblad et al. (2004): (i) subjective memory impairment corroborated by the subject and an informant; (ii) objective memory performance documented by an AVLT-DR score ≤1.5 standard deviation (SD) of age- and education-adjusted norms (cut-off of ≤4 correct responses on 12 items for subjects with ≥8 years of education); (iii) MMSE score of 24 or higher; (iv) CDR of 0.5; (v) no or minimal impairment in activities of daily living; and (vi) absence of dementia or insufficient dementia to meet the Neurological Disorders and Stroke–Alzheimer Disease and Related Disorders (NINCDS–ADRDA) Alzheimer’s Criteria. In addition, the controls were required to have a CDR of 0, a MMSE score ≥26, and an AVLT-DR score >4 for subjects with ≥8 years of education. It should be noted that the present aMCI includes single memory impairment or memory impairment plus at least one other cognitive domain. Exclusion criteria included mental or physical illness, such as stroke, head injury, alcoholism, Parkinson’s disease, epilepsy and depression, MRI contraindications, and prominent impairments of audition or vision.

### Magnetic Resonance Imaging Procedures

Magnetic resonance imaging scanning was performed using a 3 Tesla MRI scanner (Achieva 3.0 T Ingenia; Philips Medical Systems, Eindhoven, Netherlands) with a 32-channel head coil. All subjects were told to relax, close their eyes, and stay awake during scanning. Their ears were occluded with earplugs, and their heads were immobilized using belts and foam pads to minimize head motion. High-resolution T_1_-weighted sagittal images covering the whole brain were obtained by a 3D-magnetization prepared rapid gradient-echo sequence: repetition time (TR) = 9.8 ms; echo time (TE) = 4.6 ms; field of view (FOV) = 256 mm × 256 mm; acquisition matrix = 256 × 256; flip angle (FA) = 8°; thickness = 1.0 mm, gap = 0 mm; and number of slices = 192. Resting-state functional images, including 230 volumes, were obtained by a gradient-recalled echo-planar imaging sequence: TR = 2,000 ms; TE = 30 ms; FOV = 192 mm × 192 mm; acquisition matrix = 64 × 64; FA = 90°; thickness = 4.0 mm; gap = 0 mm; and number of slices = 35.

### Data Pre-processing

A toolbox for Data Processing and Analysis for Brain Imaging (DPABI) V2.3^1^ was used in the data pre-processing. The first 10 volumes of the scanning session were discarded due to T_1_ equilibration effects. The slice timing and realignment procedures were conducted to correct for the time differences in acquisition among slices within one volume, and the motion effects (Friston 24-parameter model) during scanning. No subject was excluded due to head motion artifacts exceeding 2° in rotation or 2 mm in transition. Then, spatially normalized into the Montreal Neurological Institute (MNI) echo-planar imaging (EPI) template using the default settings and resampling to 3 mm × 3 mm × 3 mm voxels were performed, and a Gaussian kernel of 6 mm × 6 mm × 6 mm was used to smooth these data. White matter signal, cerebrospinal fluid signal and 24 head motion parameters were removed as covariates of no interest. The resulting fMRI data were band-pass filtered (0.01–0.08 Hz), and the linear trend of time courses was removed. Finally, scrubbing was performed. Volumes with framewise displacement (FD) larger than 0.5 mm with prior 1 and later 2 volumes were deleted, and subjects with fewer than 4 min of remaining data (about 50% volumes) were excluded. After exclusion, 25 control subjects and 28 aMCI subjects remained.

### Functional Connectivity Analyses

A FC analysis was performed. Bilateral hippocampus masks were taken from the Anatomical Automatic Labeling (AAL), a digital atlas of the human brain, using a Resting State fMRI Data Analysis Toolkit (REST) 1.8 software.^2^ The hippocampus masks served as seed regions for hippocampal networks. Six-millimeter radius spheres centered at bilateral MPFC (MNI space: −4, 54, −8/4, 54, and −8) served as seed regions for bilateral MPFC networks (Unschuld et al., 2013; Elton et al., 2019; Zhao et al., 2020). For each subject, a mean time series for the hippocampus/MPFC (left and right separately) was computed as the reference time course. A cross-correlation analysis was performed between a mean signal change in a region of interest and a time series of every voxel in the remainder of the brain. A normality of the correlation coefficients was performed using Fisher’s Z-transformation. Twenty-four head motion parameters and a mean time series of global/white matter/cerebrospinal fluid signals were introduced as covariates into a random effects model.

### Volume Assessment of Gray Matter

Gray matter volume was measured using the VBM8 toolbox for SPM12. The detailed procedure was shown in the Supplementary Material.

### Statistical Analysis

#### Behavioral Data

The individual raw scores of each cognitive test (except for MMSE) were transformed to *Z* scores, as shown in the following equation:


$$Z_{i}=\frac{r_{i}-m}{S}$$

*Z*_*i*_ indicates the *Z* scores for the *i*th subject; *r*_*i*_ indicates the raw score for the *i*th subject; *m* indicates the average score of each test for the whole cohort; *S* indicates the standard deviation of test scores for the whole cohort. The neuropsychological tests were grouped into five cognitive domains, including episodic memory, executive function, language function, visual spatial function, and processing speed. The composite *Z* score of each cognitive domain was obtained by averaging the *Z* scores of relevant neuropsychological tests, according to the following divisions: episodic memory (two tests, including AVLT-DR and VR-DR), executive function (two tests, including TMT-B and Stroop-C), language function (BNT), visual spatial function (CDT), and processing speed (3 tests, including Stroop-A, Stroop-B, and TMT-A). Notably, the *Z* scores of executive function and processing speed were calculated using the reciprocal of time in Stroop tests and TMT tests. To differentiate between encoding problem and delayed recall problem in AVLT, we also analyzed AVLT-IR performance and compared AVLT-IR performance and AVLT-DR performance in the aMCI group. AVLT-IR scores were obtained by averaging the scores of the three trials of AVLT-IR, and was then transformed to *Z* scores. Before comparing AVLT-IR scores and AVLT-DR scores in the aMCI group, the scores were normed relative to the control group. A χ^2^ test was applied in the analysis of gender, and other demographic and neuropsychological data were analyzed using independent-samples *t*-tests or Mann-Whitney *U* tests. A paired-samples *t*-test was used in the comparison of AVLT-IR performance and AVLT-DR performance in the aMCI group. A correlative analysis was performed between the episodic memory scales and the executive function scales in each group. The significance was set at *P* < 0.05.

#### Group-Level Intrinsic Functional Connectivity Analysis

To determine patterns of the hippocampal networks and the MPFC networks in each group (i.e., within group analysis), spatial maps of FC were submitted to a random-effect analysis using one-sample *t*-tests. Statistical thresholds were set at a *P* < 0.01 and were corrected by a Monte Carlo simulation for multiple comparisons in the whole gray matter (voxel-wise *P* < 0.01, FWHM = 8.3 mm and a minimum cluster size of 2,592 mm^3^). The cluster size was calculated using the program REST AlphaSim in REST 1.8 with a GrayMask. To explore interactions of the hippocampal networks and the MPFC networks in the control group and the aMCI group, a general linear model was used to analyze interactions of groups (aMCI group vs. control group)-by-networks (hippocampal network vs. MPFC network) in FC data. A group × network analysis of covariance (ANCOVA) was performed, controlling for age, gender, and years of education. Then, the average FC strength of each significant cluster was extracted in each group, and *post hoc* tests were performed to compare the FC of each network with each cluster between groups. To explore differences in the hippocampal networks between the aMCI group and the control group, independent-samples *t*-tests were performed between the two groups of hippocampal network images controlling for age, gender, and years of education. To test whether the differences in the hippocampal networks could be attributed to the differences in the MPFC networks, independent-samples *t*-tests were also performed in the hippocampal networks additionally controlling for the MPFC networks. The MPFC network images were regressed out in a voxel-wise way when the independent-samples *t*-tests were performed using the REST 1.8 software. All statistical thresholds were set at an AlphaSim-corrected *P* < 0.01 determined by a Monte Carlo simulation (voxel-wise *P* < 0.01, FWHM = 8.3 mm and a minimum cluster size of 2,592 mm^3^).

#### Networks and Behavioral Significance

We explored the behavioral significance of the interaction of neural networks. A partial correlative analysis was performed between the scores of neuropsychological tests and the averaged FC values of the significant clusters, controlling for age, gender, and years of education. Thresholds were set at a *P* < 0.05.

## Results

### Demographic and Neuropsychological Data

**FIGURE 1:**
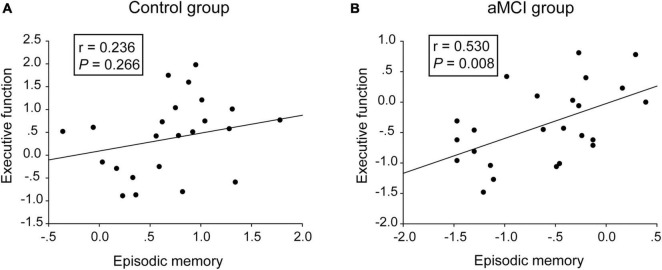
Correlative analyses between episodic memory scales and executive function scales. **(A,B)** The scores of the episodic memory scales were significantly positively correlated with the executive function scales in the aMCI group, rather than in the control group. The statistical thresholds were set at a *P* < 0.05.

As shown in Table 1, no significant difference of demographic characteristics, language function or visual spatial function was found between the aMCI group and the control group. Compared with the control group, the aMCI group showed deficits in CDR, MMSE, episodic memory (i.e., the AVLT-DR scores and the VR-DR scores), executive function (i.e., the TMT-B scores and the Stroop-C scores), encoding (i.e., AVLT-IR scores), and processing speed (i.e., Stroop-A, Stroop-B, and TMT-A). Normed relative to the control group, the AVLT-DR performance of the aMCI group was more impaired than the AVLT-IR performance (Supplementary Figure 1), suggesting that delayed recall was more impaired than encoding in the aMCI group. Moreover, the episodic memory scores were significantly positively correlated with the executive function scores in the aMCI group (*r* = 0.530, *P* = 0.008), not in the control group (*r* = 0.236, *P* = 0.266) (Figure 1). Notably, no significant difference in gray matter volume was shown between the aMCI group and the control group (Supplementary Table 1).

**TABLE 1:** Demographic and neuropsychological data.

**Items**	**Controls (*n* = 25)**	**aMCI (*n* = 28)**	**t or χ ^2^**	***P***
Age (years)	63.08 ± 5.94	66.75 ± 9.63	−1.69	0.098
Education (years)	12.16 ± 3.45	11.36 ± 3.12	–	0.385
Gender (male: female)	12:13	9:19	1.39	0.239
CDR	0	0.5	–	–
MMSE	29.28 ± 0.79	27.11 ± 1.13	–	<0.001
Episodic memory	0.72 ± 0.51	−0.62 ± 0.55	8.95	<0.001
AVLT-DR	0.88 ± 0.66	−0.78 ± 0.43	10.81	<0.001
VR-DR	0.57 ± 0.87	−0.48 ± 0.84	4.4	<0.001
Executive function	0.39 ± 0.83	−0.41 ± 0.64	3.84	<0.001
Stroop-C	0.33 ± 0.96	−0.32 ± 0.94	2.54	0.018
TMT-B	0.45 ± 1.06	−0.45 ± 0.71	3.54	0.001
Encoding				
AVLT-IR	0.57 ± 1.01	−0.49 ± 0.69	4.5	<0.001
Language function				
BNT	0.26 ± 0.90	−0.25 ± 1.01	2.04	0.051
Visual-spatial function				
CDT	0.24 ± 0.89	−0.24 ± 1.03	1.71	0.062
Processing speed	0.33 ± 0.78	−0.33 ± 0.79	3.01	0.004
Stroop-A	0.28 ± 0.83	−0.27 ± 1.09	2.02	0.049
Stroop-B	0.38 ± 0.98	−0.37 ± 0.89	2.88	0.006
TMT-A	0.34 ± 0.89	−0.34 ± 1.01	2.52	0.015

*Values are presented as mean ± stand deviation (SD). Fisher’s Z-transformation was performed in episodic memory, executive function, encoding, language function, visual spatial function, and processing speed data.*

*Independent-samples *t*-tests were applied in the analyses of age, episodic memory, executive function, encoding data, language function, visual spatial function, and processing speed. χ^2^ test was applied in the analysis of gender. Mann-Whitney *U* tests were applied in the analyses of education and MMSE scores.*

*aMCI, amnestic mild cognitive impairment; AVLT-IR, Auditory Verbal Learning Test-immediate recall; AVLT-DR, Auditory Verbal Learning Test-delayed recall; BNT, Boston Naming Test; CDR, Clinical Dementia Rating; CDT, Clock Drawing Test; MMSE, Mini Mental State Examination; Stroop-A, -B and -C, Stroop Color and Word Tests-A, -B and -C; TMT-A and -B, Trail Making Tests-A and -B; VR-DR, Visual Replication-delayed recall.*

### Group-Level Intrinsic Functional Connectivity

#### Within Each Group

**FIGURE 2:**
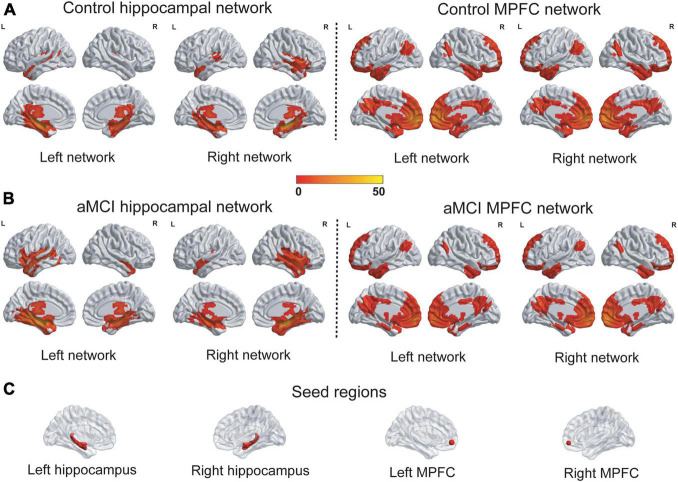
The patterns of the hippocampal networks and the MPFC networks. **(A,B)** The hippocampal networks and the MPFC networks were identified in the control group and the aMCI group. A qualitative visual inspection of the FC revealed that the majority of clusters were highly consistent between the groups. The statistical thresholds were set at a corrected *P* < 0.01, which were determined by a Monte Carlo simulation. The color bar is presented with *t* values. **(C)** The seed regions for networks were depicted. L, left; R, right; MPFC, medial prefrontal cortex; FC, Functional connectivity.

The hippocampal networks and the MPFC networks were identified in the control group and the aMCI group (Figure 2). A qualitative visual inspection revealed that the majority of clusters were highly consistent between the groups. As shown in Supplementary Table 2, the hippocampal networks encompassed a number of specific distributed cortical and subcortical regions in the temporal, posterior cingulate and lateral parietal regions, and the patterns of the networks were consistent with those in previous studies using the whole hippocampus as seeds for networks (Dickerson and Eichenbaum, 2010; Song et al., 2018; Zhu et al., 2018) but were sparser than those in a study using spheres in the anterior hippocampus as seeds (Vincent et al., 2006). The MPFC networks extensively encompassed cortical and subcortical regions in the frontal, temporal, parietal and cingulate regions, and the patterns of networks were consistent with those in previous studies (Elliott, 2003; Elton et al., 2019).

#### Between the Groups

**FIGURE 3:**
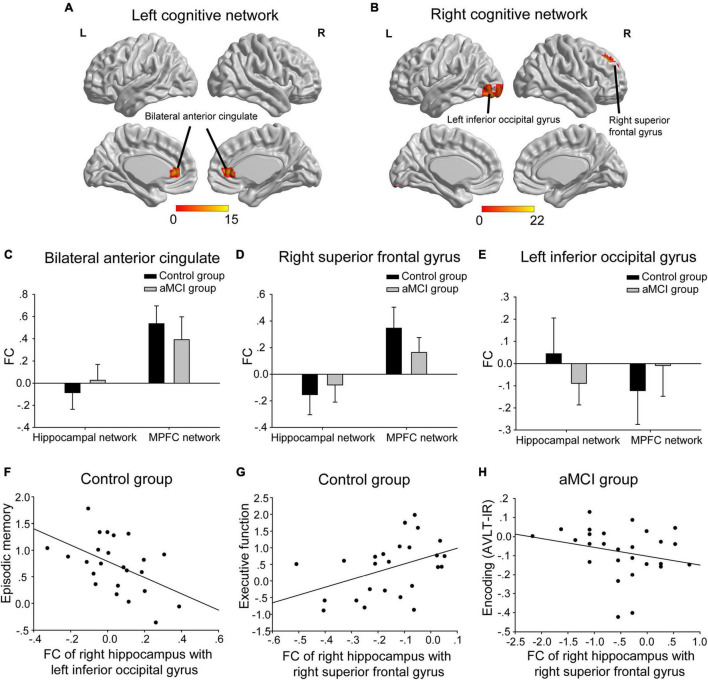
The interactions of groups-by-networks. **(A)** The interactions of the group-by-left cognitive networks were mainly in bilateral anterior cingulate. **(B)** The interactions of the group-by-right cognitive networks were mainly in the right superior frontal gyrus and the left inferior occipital gyrus. The statistical thresholds were set at a corrected *P* < 0.01, which was determined by a Monte Carlo simulation. The color bars are presented with *F* values. **(C,D)** In both bilateral anterior cingulate and the right superior frontal gyrus, the FC of the hippocampal network was higher in the aMCI group than in the control group, whereas the FC of the MPFC network was lower in the aMCI group than in the control group. **(E)** In the left inferior occipital gyrus, the FC of the hippocampal network was lower in the aMCI group than in the control group, whereas the FC of the MPFC network was higher in the aMCI group than in the control group. **(F)** The FC of the right hippocampus with the left inferior occipital gyrus was negatively associated with episodic memory scales in the control group. **(G)** The FC of the right hippocampus with the right superior frontal gyrus was positively associated with executive function scales in the control group. **(H)** The FC of the right hippocampus with the right superior frontal gyrus was negatively associated with encoding (i.e., AVLT-IR) scales in the aMCI group. The above FC value in each significant cluster was the averaged FC strength in each cluster. L, left; R, right; FC, functional connectivity; AVLT-IR, auditory verbal learning test-immediate recall.

(1) The interactions of group (aMCI group vs. control group)-by-network (hippocampal network vs. MPFC network) were mainly in bilateral anterior cingulate, the right superior frontal gyrus and the left inferior occipital gyrus (Table 2 and Figures 3A,B). A *post hoc* analysis showed that in both bilateral anterior cingulate and the right superior frontal gyrus, the FC of the hippocampal network was higher in the aMCI group than in the control group, whereas the FC of the MPFC network was lower in the aMCI group than in the control group (Figures 3C,D). In the left inferior occipital gyrus, conversely, the FC of the hippocampal network was lower in the aMCI group than in the control group, whereas the FC of the MPFC network was higher in the aMCI group than in the control group (Figure 3E). In the correlative analyses between the neuropsychological test scores and the interaction of hippocampal networks and MPFC networks, the FC of the right hippocampus with the left inferior occipital gyrus and the right superior frontal gyrus were significantly associated with episodic memory and executive function in the control group, respectively (*r* = −0.493, *P* = 0.014 and *r* = 0.427, *P* = 0.033) (Figures 3F,G). Furthermore, the FC between the right hippocampus and the right superior frontal gyrus was significantly associated with the encoding performance in the aMCI group (*r* = −0.548, *P* = 0.005) (Figure 3H).

**TABLE 2:** Interactions between disease and cognitive networks.

**Networks**	**Brain regions with group differences**	**BA**	**Peak MNI coordinates x, y, z (mm)**	**Peak *F* value**	**Cluster size (mm^3^)**
**Left cognitive networks**					
	Bilateral anterior cingulate	10, 11, 24, 32	−3, 33, 3	12.69	4,563
**Right cognitive networks**					
	Right superior frontal gyrus	8, 9, 32	−42, −84, −21	19.34	6,588
	Left inferior occipital gyrus	17, 18, 19	18, 33, 42	21.39	4,158

*The thresholds were set at a corrected *P* < 0.01, determined by Monte Carlo simulation for multiple comparisons.*

*BA, Brodmann’s area; MNI, Montreal Neurological Institute.*

**FIGURE 4:**
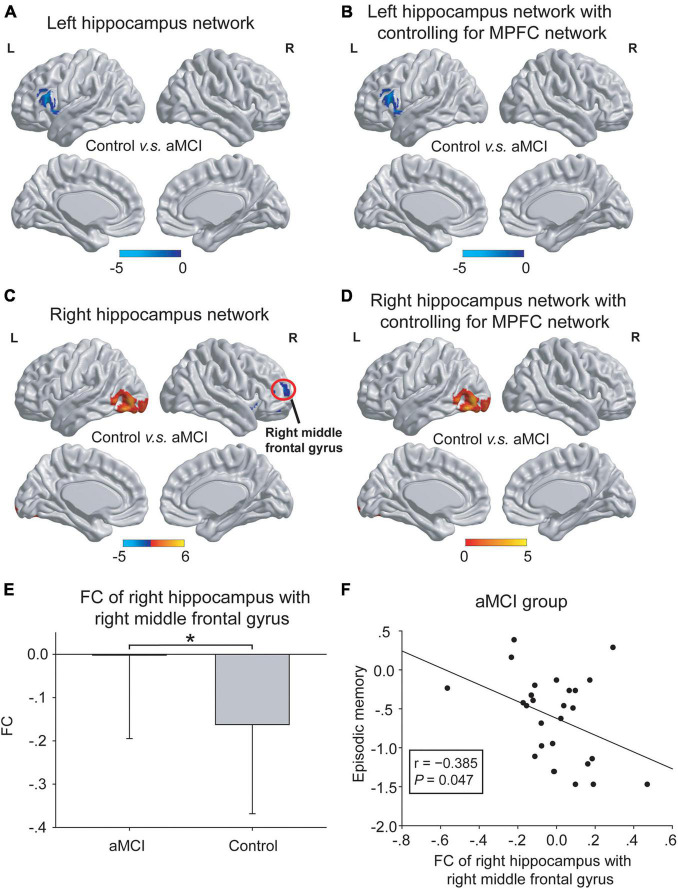
The differences in hippocampal networks between the control group and the aMCI group. **(A,B)** In the left hippocampal network, compared with the control group, the aMCI group showed increased FC in the left frontal cortex (blue regions) both with and without controlling for the MPFC network. No significant influence of the MPFC network onto the left hippocampal network was shown. **(C–E)** In the right hippocampal network, compared with the control group, the aMCI group showed decreased FC in the left occipital cortex (red regions) both with and without controlling for the MPFC network. Notably, the aMCI group showed increased FC in the right middle frontal gyrus [red circle in panel **(C)**] only without controlling for the MPFC network [also shown in panel **(E)**]. The statistical thresholds were set at a corrected *P* < 0.01, which was determined by a Monte Carlo simulation. The color bars are presented with *t* values. **(F)** The increased FC of the right hippocampus with the right middle frontal gyrus was negatively associated with episodic memory scales in the aMCI group. The above FC value in each significant cluster was the averaged FC strength in each cluster, and the error bars in panel **(E)** represent standard deviations of FC values in each group. *P < 0.05. L, left; R, right; FC, functional connectivity; MPFC, medial prefrontal cortex.

(2) Abnormal hippocampal connectivity maps were observed in the aMCI group (Figure 4). In the left hippocampal network, compared with the control group, the aMCI group showed increased FC of the left hippocampus in the prefrontal cortex with or without controlling for the MPFC network (Figures 4A,B). That is, no significant influence of the MPFC network on the left hippocampal network was found. In the right hippocampus network, compared to the control group, the aMCI group showed decreased FC of the right hippocampus in the occipital cortex with or without controlling for the MPFC network. Notably, the aMCI group showed increased FC of the right hippocampus with the right middle frontal gyrus without controlling for the MPFC network, and this difference in the hippocampus network disappeared when controlling for the MPFC network (Figures 4C–E). Interestingly, the increased FC in the right middle frontal gyrus was negatively correlated with the episodic memory performance in the aMCI group (*r* = −0.385, *P* = 0.047) (Figure 4F).

## Discussion

This study investigated the relationship between episodic memory and executive function on the one hand and the association between the hippocampal networks and the MPFC networks on the other hand. Two major findings were as follows: (i) the episodic memory impairment in aMCI may be partially due to the executive function impairment; (ii) the MPFC network was selectively involved in regulating the right hippocampal FC with the right prefrontal cortex and the regulation was associated with episodic memory in aMCI subjects. These findings extend our understanding of the neurophysiologic mechanisms involved in the executive decline hypothesis in aMCI.

Our neuropsychological results showed both significantly impaired episodic memory and executive function in aMCI subjects. The role of executive function in cognitive aging is related to memory and has been discussed in the “Executive Decline Hypothesis” (Salthouse et al., 2003; Lee et al., 2012). The hypothesis suggests that aging is not the core factor driving memory decline. Instead, executive function is a more important factor. Episodic memory is the predominant impairment in aMCI, and executive function is commonly impaired in AD patients and, to a lesser extent, in aMCI patients (Aretouli et al., 2013; Yoon et al., 2013), which appears contradictory to the hypothesis. However, it is plausible that more complex neurophysiologic mechanisms are involved in the progression of aMCI compared with normal aging. In the present study, the linear relationship between episodic memory and executive function in aMCI subjects suggests that this hypothesis may be associated with progressive cognitive impairments in aMCI subjects.

A novel finding in our study was the identification of the interaction between the hippocampal networks and the MPFC networks in aMCI subjects and control subjects. Previous studies have found disrupted patterns in hippocampal networks (Risacher et al., 2013; Liu et al., 2016; Ye et al., 2017) and executive control networks (Liu et al., 2012; Wu et al., 2014) in subjects with mild cognitive impairment. Decreased FC between the hippocampus and the MPFC has been shown in subjects with mild cognitive impairment (Liu et al., 2016; Berron et al., 2020). In contrast, the present study showed the direct interaction between the hippocampal networks and the MPFC networks in control subjects and aMCI subjects. The *post hoc* analysis suggested that although the FC in the three regions related to the interaction was altered in the aMCI group as compared with the control group, the patterns of the alterations were opposite between the hippocampal networks and the MPFC networks. In the aMCI group, the FC of the hippocampal networks was increased in anterior cortices (bilateral anterior cingulate and the right superior frontal gyrus) but was decreased in the posterior cortex (the left inferior occipital gyrus). In contrast, the FC of the MPFC networks was decreased in anterior cortices but was increased in the posterior cortex in the aMCI group, suggesting the different roles of these regions in the hippocampal networks and the MPFC networks. Furthermore, the FC in the regions were significantly associated with executive function and episodic memory encoding and retrieval. Thus, the hippocampal networks could support cognitive function by connecting with these regions in different ways according to cognitive status (aMCI or cognitively normal). Actually, the prefrontal cortex, the core of the MPFC networks, plays a critical role in successful executive processing (Girotti et al., 2018) and episodic memory encoding and retrieval (Kragel et al., 2017). And the medial temporal lobe, the core of the hippocampal networks, is mainly involved in episodic memory encoding and subsequent retrieval performance (Matthews, 2015). The present findings partially explain the association between episodic memory impairment and executive function impairment at the functional network level. It’s worth noting that the hippocampal networks or the MPFC networks are not entirely specific to episodic memory or executive function, respectively. Thus, the link between episodic memory and executive function should not be equal to that between the hippocampal networks and the MPFC networks.

The interaction between the hippocampus and the prefrontal cortex in recognition memory retrieval has been well documented. The prefrontal cortex has also been linked with cognitive control processes, such as inhibition (Simons and Spiers, 2003). In the present study, the MPFC network was associated with a right lateralization of hyper-connectivity inhibition in the hippocampal network, and the right hippocampal FC with the right frontal cortex was negatively correlated with the episodic memory performance in aMCI subjects. The right lateralization was in line with previous studies. Anderson et al. (2004) revealed that the right prefrontal cortex activation corresponded to a decrement in hippocampal activity, suggesting that suppression of unwanted memories required prefrontal cortex recruitment to disengage hippocampal processing. Another study observed that temporary inhibition of the right prefrontal cortex enhanced memory performance in aMCI subjects. This finding suggested a cognitive benefit when there was reduced activity in this region during recognition memory tasks in patients with memory impairments (Turriziani et al., 2012). Similarly, the present study found that the right hippocampal FC with the right superior frontal gyrus was negatively correlated with the encoding performance in the aMCI group. In other words, higher hippocampal FC with the frontal region was associated with poorer encoding performance in aMCI subjects. This relationship could be explained with aberrant brain activation related to worse performance (Bakker et al., 2012) or pathology- or age-related dedifferentiation of brain activities (Park et al., 2001; Burianova et al., 2013). Furthermore, the negative correlation between the right hippocampal FC with the occipital cortex and memory performance in the control group could also be explained with age-related dedifferentiation, which has been often shown in the occipital cortex (Kleemeyer et al., 2017; Berron et al., 2018). On the other hand, the right hippocampal FC with the right superior frontal gyrus was positively correlated with executive function in the control group, that is, higher hippocampal FC with the frontal region was associated with better executive function in control subjects. According to a conceptual model of cognitive aging named “the scaffolding theory of aging and cognition,” the level of cognitive function is a consequence of neural degradation, compensatory neural processes, and life-course factors (Reuter-Lorenz and Park, 2014). Correlative analysis between brain activities and cognitive function based on cross-sectional data might show conflicting results. Longitudinal data would be helpful to investigating the causal relationship. It should be noted that the present findings suggested lateralized contributions of the MPFC networks to the hippocampal networks, rather than lateralized effects of the MPFC networks or the hippocampal networks on cognitive function.

The hippocampal networks in the present study were identified using the whole hippocampus masks as seed regions. Differential FC of the anterior and posterior hippocampus has been shown by previous studies; The anterior hippocampus is more strongly connected with orbitofrontal and temporal regions, whereas the posterior hippocampus is more strongly connected with lateral parietal and lateral prefrontal regions (Adnan et al., 2016; Frank et al., 2019). Similarly, differential structural connectivity of the anterior and posterior hippocampus has been identified by using diffusion-weighted imaging-based parcelation (Adnan et al., 2016). These differential functional and structural connectivity contributes to functional specializations along the long axis of the hippocampus, i.e., generalized, coarse representations in anterior hippocampus and local, detailed representations in posterior hippocampus (Poppenk et al., 2013; Frank et al., 2019). The locus of hippocampal damage in the anterior or posterior hippocampus is related to specific clinical manifestations of various neurological and psychiatric disorders (Strange et al., 2014). Future fMRI studies should consider the different roles of the anterior and posterior hippocampus, and exploring the relationship of the MPFC networks with the anterior and posterior hippocampal networks may provide more evidence related to the executive decline hypothesis.

There are several issues to be addressed. First, the recruitment diagnosis of aMCI subjects was based on clinical criteria. Consolidation deficit, an important characteristic that discriminates AD from other dementias (Economou et al., 2016), was not measured in the present study. Some of the aMCI subjects may not be indeed prodromal AD patients and may have “contaminated” the sample with non-AD cases. Second, the results of correlation analyses between FC and cognitive performance were not corrected by the Bonferroni correction principle. Third, a gray matter volume correction was not performed for the functional brain networks. The differences in functional networks might be potentially influenced by brain atrophy, although no significant difference of gray matter volume was shown between groups. Finally, the seeds for the MPFC networks in the present study were more ventral and anterior than brain regions commonly activated during executive function tasks as shown by a meta-analysis of 193 task-state fMRI studies (Niendam et al., 2012), despite the seeds in the present study were chosen based on published findings and were similar with those in previous studies (Unschuld et al., 2013; Elton et al., 2019; Oh et al., 2019; Zhao et al., 2020). Although the MPFC serves as a central component of systems involved in executive function, there are also other regions (e.g., dorsolateral prefrontal cortex) closely related to executive function. The selection of seeds may influence the results. Thus, the present findings should be treated with caution.

## Conclusion

The MPFC networks interacted with the hippocampal networks and regulated the right hippocampal network in aMCI subjects, and these effects were associated with episodic memory deficits in aMCI subjects. The present findings might support executive decline hypothesis, and future researches may validate the effectiveness of executive function interventions in AD-spectrum patients.

## Data Availability Statement

The raw data supporting the conclusions of this article will be made available by the authors, without undue reservation.

## Ethics Statement

The studies involving human participants were reviewed and approved by the Drum Tower Hospital Research Ethics Committee. The patients/participants provided their written informed consent to participate in this study.

## Author Contributions

FB and HZ: conceptualization, project administration, funding acquisition, and writing—review and editing. YX: conceptualization, project administration, supervision, and funding acquisition. QY: writing—original draft, formal analysis, funding acquisition, and methodology. HC, RL, RQ, CL, and ML: data curation and investigation. All authors approved the final version of the manuscript.

## Conflict of Interest

The authors declare that the research was conducted in the absence of any commercial or financial relationships that could be construed as a potential conflict of interest.

## Publisher’s Note

All claims expressed in this article are solely those of the authors and do not necessarily represent those of their affiliated organizations, or those of the publisher, the editors and the reviewers. Any product that may be evaluated in this article, or claim that may be made by its manufacturer, is not guaranteed or endorsed by the publisher.

## Footnotes

1
http://rfmri.org/DPABI

2
http://restfmri.net/forum/index.php
