# Supplementary material for: Lateralized Contributions of Medial Prefrontal Cortex Network to Episodic Memory Deficits in Subjects With Amnestic Mild Cognitive Impairment
Source: Front Aging Neurosci. 2021 Nov 17;13:756241. doi: 10.3389/fnagi.2021.756241 (PMC8635729; doi:10.3389/fnagi.2021.756241)
Supplement: Supplementary file 2 [file Data_Sheet_2.docx]

Supplementary Material

# Supplementary Methods

**1.1 Volume assessment of gray matter**

Gray matter volume was measured using the VBM8 toolbox for SPM12. First, the T1 images were segmented into gray matter, white matter and cerebrospinal fluid. Second, the segmented images were normalized to the MNI template using a non-linear and affine spatial normalization and re-sampled to a voxel size of 1.5 × 1.5 × 1.5 mm. Third, the segmented images were incorporated to compensate for the effect of spatial normalization using Jacobian modulation. Fourth, the extracted gray matter, white matter and cerebrospinal fluid sets were smoothed with an 8-mm full width at half maximum Gaussian filter to decrease the effects of individual variation in gyral anatomy and to increase the signal-to-noise ratio. Finally, gray matter volume, white matter volume and cerebrospinal fluid volume were obtained in each subject.

# Supplementary Figures and Tables

**2.1 Supplementary Figure 1**





**Supplementary Figure 1** Comparison of AVLT-DR performance and AVLT-IR performance in the aMCI group. Based on AVLT performance from the control group, normative Z-scores were calculated for AVLT-DR and AVLT-IR in the aMCI group, respectively. AVLT performance in the aMCI group revealed greater impairment in AVLT-DR than that in AVLT-IR. The error bars represent standard deviations of Z-scores. * *P* < 0.05. Abbreviations: aMCI, amnestic mild cognitive impairment; AVLT-IR, Auditory Verbal Learning Test-immediate recall; AVLT-DR, Auditory Verbal Learning Test-delayed recall.

## 2.2 Tables

| **Supplementary Table 1 Brain volume data** | | | | |  |
| --- | --- | --- | --- | --- | --- |
|  |  |  |  |  |  |
| Items | Controls (*n* = 25) | aMCI (*n* = 28) | t | *P* |  |
| **Gray matter volume (mL)** | 540.38±48.00 | 521.39±49.65 | 1.55 | 0.125 |  |
| Values are presented as mean ± stand deviation (SD). Independent-samples t-test was applied in the analyses. Abbreviations: aMCI, amnestic mild cognitive impairment. | | | | |  |

| **Supplementary Table 2 Hippocampal networks and MPFC networks in each group** | | | | | | |  |
| --- | --- | --- | --- | --- | --- | --- | --- |
|  |  |  |  |  |  |  |  |
| **Groups** | **Networks** | **Clusters** | **BA** | **Peak MNI coordinates x, y, z (mm)** | **Peak F value** | **Cluster size (mm^3^)** |  |
| **Control group** | | | | | | |  |
|  | Left hippocampal network | | | | | |  |
|  |  | Bilateral parahippocampal gyrus, thalamus, fusiform, superior temporal gyrus, posterior cingulate, precuneus and corpus callosum, and left middle/inferior temporal gyrus | 20, 28, 36, 34, 37, 35, 30, 21, 38, 27, 13, 23, 19, 29. 41, 47, 31 | −33, −18, −15 | 24.71 | 172611 |  |
|  | Right hippocampal network | | | | | |  |
|  |  | Bilateral parahippocampal gyrus, thalamus, middle temporal gyrus, fusiform, posterior cingulate, corpus callosum and precuneus, and right superior temporal gyrus, inferior frontal gyrus and insula | 38, 21, 20, 13, 47, 28, 22, 34, 36, 30, 37, 35, 23, 29, 19, 41, 27 | 33, −12, −18 | 23.04 | 187488 |  |
|  |  | Left superior/middle temporal gyrus | 42, 22 | −63, −30, 12 | 4.62 | 4698 |  |
|  | Left MPFC network | | | | | |  |
|  |  | Bilateral superior/medial/middle/inferior frontal gyrus, cingulum, precuneus, middle/inferior temporal gyrus, caudate, rectus, parahippocampal gyrus, cuneus, corpus callosum, fusiform, putamen, amygdala, insula and hippocampus | 11, 10, 9, 8, 31, 47, 32, 21, 38, 7, 25, 24, 20, 28, 34, 6, 23, 35, 30, 36, 29 | 0, 54, −9 | 42.51 | 311607 |  |
|  |  | Right angular gyrus, middle/superior temporal gyrus, supramarginal gyrus and middle occipital gyrus | 39, 40, 22 | 54, −66, 30 | 6.70 | 8343 |  |
|  |  | Left angular gyrus, middle/superior temporal gyrus, supramarginal gyrus, middle occipital gyrus, inferior parietal lobule and precuneus | 39, 40, 19, 22 | −42, −75, 39 | 7.89 | 12123 |  |
|  | Right MPFC network | | | | | |  |
|  |  | Bilateral superior/medial/middle/inferior frontal gyrus, cingulum, precuneus, middle/inferior/superior temporal gyrus, caudate, rectus, parahippocampal gyrus, cuneus, corpus callosum, fusiform, putamen, amygdala, insula and hippocampus | 11, 10, 9, 8, 31, 47, 32, 21, 38, 7, 25, 24, 20, 28, 34, 6, 23, 35, 30, 36, 29 | 0, 54, −9 | 45.15 | 315360 |  |
|  |  | Right angular gyrus, middle/superior temporal gyrus, supramarginal gyrus and middle occipital gyrus | 39, 40, 22 | 54, −66, 30 | 7.62 | 10449 |  |
|  |  | Left angular gyrus, middle/superior temporal gyrus, supramarginal gyrus, middle occipital gyrus, inferior parietal lobule and precuneus | 39, 40, 19, 22 | −45, −75, 39 | 7.77 | 12231 |  |
| **aMCI group** | | | | | | |  |
|  | Left hippocampal network | | | | | |  |
|  |  | Bilateral parahippocampal gyrus, superior/middle/inferior temporal gyrus, fusiform, insula, thalamus, posterior cingulate, putamen, precuneus, corpus callosum, lingual gyrus and left middle/inferior temporal gyrus and inferior frontal gyrus | 21, 13, 38, 20, 47, 22, 28, 30, 19, 37, 36, 34, 41, 35, 25, 18, 29, 45, 43, 23 | −30, −24, −9 | 21.33 | 259794 |  |
|  | Right hippocampal network | | | | | |  |
|  |  | Bilateral parahippocampal gyrus, superior/middle temporal gyrus, thalamus, fusiform, posterior cingulate, corpus callosum and precuneus, and right inferior temporal gyrus, inferior frontal gyrus, precentral gyrus and insula | 21, 13, 38, 22, 20, 47, 28, 36, 41, 34, 30, 19, 35, 37, 42, 11, 40, 25, 6, 43 | 30, −18, −12 | 27.71 | 261414 |  |
|  | Left MPFC network |  |  |  |  |  |  |
|  |  | Bilateral superior/medial/middle/inferior frontal gyrus, cingulum, precuneus, middle/inferior temporal gyrus, caudate, rectus, parahippocampal gyrus, cuneus, corpus callosum, fusiform, putamen, amygdala, insula and hippocampus | 10, 11, 9, 31, 8, 32, 47, 7, 21, 38, 20, 25, 24, 23, 30, 6, 34, 35, 28, 36, 29, 13 | −3, 54, −9 | 71.69 | 282609 |  |
|  |  | Right angular gyrus, superior/middle temporal gyrus, middle occipital gyrus and supramarginal gyrus | 39, 22, 40 | 57, −69, 33 | 6.54 | 4482 |  |
|  |  | Left angular gyrus, middle/superior temporal gyrus, supramarginal gyrus, middle occipital gyrus, precuneus and inferior parietal lobule | 39, 19 | −48, −69, 33 | 6.74 | 8046 |  |
|  | Right MPFC network | | | | | |  |
|  |  | Bilateral superior/medial/middle/inferior frontal gyrus, cingulum, precuneus, middle/inferior/superior temporal gyrus, caudate, rectus, parahippocampal gyrus, cuneus, corpus callosum, fusiform, putamen, amygdala, insula and hippocampus | 10, 11, 9, 8, 32, 47, 21, 38, 20, 25, 24, 6, 28, 34, 35, 36, 13 | 3, 54, −9 | 46.78 | 278856 |  |
|  |  | Right angular gyrus, middle/superior temporal gyrus, supramarginal gyrus and middle occipital gyrus | 39, 22, 40 | 57, −69, 33 | 6.81 | 5724 |  |
|  |  | Left angular gyrus, middle/superior temporal gyrus, middle occipital gyrus, precuneus and inferior parietal lobule | 39, 19 | −48, −69, 33 | 6.50 | 6696 |  |
| The thresholds were set at a corrected *P* < 0.01, determined by Monte Carlo simulation for multiple comparisons. Abbreviations: BA, Brodmann’s area; MNI, Montreal Neurological Institute. | | | | | | |  |
|  |  |  |  |  |  |  |  |
|  |  |  |  |  |  |  |  |
